# Operando Synchrotron X‐Ray Absorption Spectroscopy: A Key Tool for Cathode Material Studies in Next‐Generation Batteries

**DOI:** 10.1002/advs.202414480

**Published:** 2025-01-24

**Authors:** Yameng Fan, Xin Wang, Guyue Bo, Xun Xu, Khay Wai See, Bernt Johannessen, Wei Kong Pang

**Affiliations:** ^1^ Institute for Superconducting & Electronic Materials (ISEM) Faculty of Engineering and Information Sciences (EIS) University of Wollongong Wollongong NSW 2500 Australia; ^2^ Australian Synchrotron Australian Nuclear Science and Technology Organization Clayton VIC 3168 Australia

**Keywords:** battery, cathode, synchrotron technique, X‐ray absorption spectroscopy

## Abstract

Rechargeable batteries are central to modern energy storage systems, from portable electronics to electric vehicles. The cathode material, a critical component, largely dictates a battery's energy density, capacity, and overall performance. This review focuses on the application of operando X‐ray absorption spectroscopy (XAS) to study cathode materials in Li‐ion, Na‐ion, Li–S, and Na–S batteries. Operando XAS provides real‐time insights into the local electronic structure, oxidation states, and coordination environments, which are crucial for understanding complex electrochemical processes, such as redox reactions, phase transitions, and structural degradation. The review highlights the strengths of hard and soft XAS techniques in probing transition metal (TM) and anionic redox processes, particularly in layered oxide cathodes, where reversible oxygen redox and TM behavior are pivotal. The role of operando XAS is also explored in analyzing conversion‐type S‐based cathodes, where it helps unravel the intricate reaction mechanisms. Furthermore, the review addresses the challenges of in situ cell design for operando XAS, especially under ultrahigh vacuum conditions for soft XAS. By discussing recent advancements and future directions, this review underscores the critical role of operando XAS in driving innovation and improving the design and performance of next‐generation battery technologies.

## Graphical abstract

Operando X‐ray absorption spectroscopy (XAS) provides real‐time insights into cathode materials for rechargeable batteries, capturing dynamic redox mechanisms, structural evolution, and interfacial behaviors. This review highlights the unique advantages and challenges of operando XAS for Li‐ion, Na‐ion, and Li/Na–S systems, bridging scientific understanding, and advanced battery design.

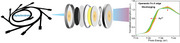

## 1 Introduction

Rechargeable batteries have become indispensable in modern society, powering a wide range of applications from portable electronics to electric vehicles and large‐scale energy storage systems.^[^ ^1^, ^2^, ^3^ ^]^ Among the components of a battery, the cathode material is particularly crucial, as it largely determines the energy density, capacity, and overall performance.^[^ ^4^ ^]^ Over the past few decades, substantial progress has been made in developing advanced cathode materials for lithium‐ion (Li‐ion), sodium‐ion (Na‐ion), lithium–sulfur (Li–S), and sodium–sulfur (Na–S) batteries.^[^ ^5^ ^]^ However, despite these advancements, the complex electrochemical processes that occur within these materials—such as redox reactions, phase transitions, and structural changes—are still not fully understood, which has posed significant challenges to optimizing battery performance.

In Li‐ion and Na‐ion batteries, the cathode materials primarily consist of alkali metal oxides, including layered oxides, polyanionic oxides, and spinel oxides.^[^ ^6^ ^]^ These materials, along with Prussian blue/white analogues for Na‐ion batteries, operate through intercalation and deintercalation mechanisms for Li and Na ion storage, with charge compensation primarily facilitated by transition metal (TM) redox reactions. However, major challenges persist, including phase transitions and structural degradation that result from the continuous insertion and extraction of alkali ions. To address these challenges, X‐ray absorption spectroscopy (XAS) has emerged as a powerful tool, providing critical insights into the underlying mechanisms by probing both TM *L*‐edge and *K*‐edge, revealing the origin of charge transfer, identifying redox processes, determining working potentials, and tracking local geometric and bond variations.^[^ ^7^ ^]^ Achieving a deeper understanding of these processes requires advanced characterization techniques, particularly high‐energy, tunable synchrotron‐based XAS.

In recent years, the exploration of reversible anionic redox reactions has garnered significant attention. Lattice oxygen in layered oxide cathodes has been identified as an additional redox center, contributing to increased capacity in batteries.^[^ ^8^ ^]^ For example, Li‐rich layered cathodes can achieve exceptionally high capacities (>250 mAh g⁻¹) by utilizing both cationic (Ni and Co) and lattice oxygen redox activities.^[^ ^9^ ^]^ However, the primary debate surrounding oxygen redox centers focuses on the nature of the oxidation products,^[^ ^10^, ^11^, ^12^ ^]^ which determine whether the reaction is reversible. In this context, soft X‐ray techniques that probe the O *K*‐edge are indispensable for investigating oxidized oxygen species and their role in battery performance.

Unlike intercalation‐type cathodes, the charge compensation in S‐based cathodes for Li–S and Na–S batteries involves conversion reactions, where sulfur undergoes transitions between elemental sulfur, polysulfides, and Li₂S/Na₂S.^[^ ^13^ ^]^ These sulfur‐based intermediates are typically amorphous or poorly crystalline and coexist within the cathode during electrochemical cycling, making them difficult to distinguish using conventional techniques, such as X‐ray diffraction (XRD) or surface‐sensitive X‐ray photoelectron spectroscopy (XPS). In contrast, XAS is highly sensitive to specific elements, enabling the identification and quantitative analysis of sulfur intermediates, thus providing a more comprehensive understanding of the reaction mechanisms in S‐based cathodes.

While traditional characterization techniques have been extensively reviewed in the literature^[^ ^7^, ^14^, ^15^, ^16^ ^]^ and remain valuable, they often fall short in capturing the dynamic and transient phenomena crucial to battery performance, particularly at the local structural level. Operando synchrotron XAS, however, has proven to be a powerful tool in this regard.^[^ ^17^ ^]^ Operando XAS allows for real‐time observation of the local electronic structure, oxidation states, and coordination environments of specific elements within cathode materials under actual operating conditions.^[^ ^18^ ^]^ By providing element‐specific information with high temporal resolution, operando XAS offers unprecedented insights into the mechanisms driving battery performance degradation.

It is important to clarify the distinction between operando and in situ measurements. While in situ measurements analyze battery materials under controlled conditions (e.g., specific voltages or currents) without directly monitoring electrochemical performance, operando measurements are conducted during real‐time operation, thereby allowing simultaneous correlation of material changes with electrochemical behavior.

This review paper focuses on the application of operando XAS to a variety of cathode materials across different rechargeable battery systems, including Li‐ion, Na‐ion, Li–S, and Na–S batteries. It explores the strengths and limitations of hard and soft XAS techniques and discusses their roles in elucidating redox chemistry, structural evolution, and interfacial phenomena in these materials. By highlighting recent advancements and identifying future directions, this review underscores the critical importance of operando XAS in driving innovation and advancing the next generation of energy storage technologies.

## 2 X‐Ray Absorption Spectroscopy

### 2.1 Why Choose Synchrotron XAS

**Figure 1:**
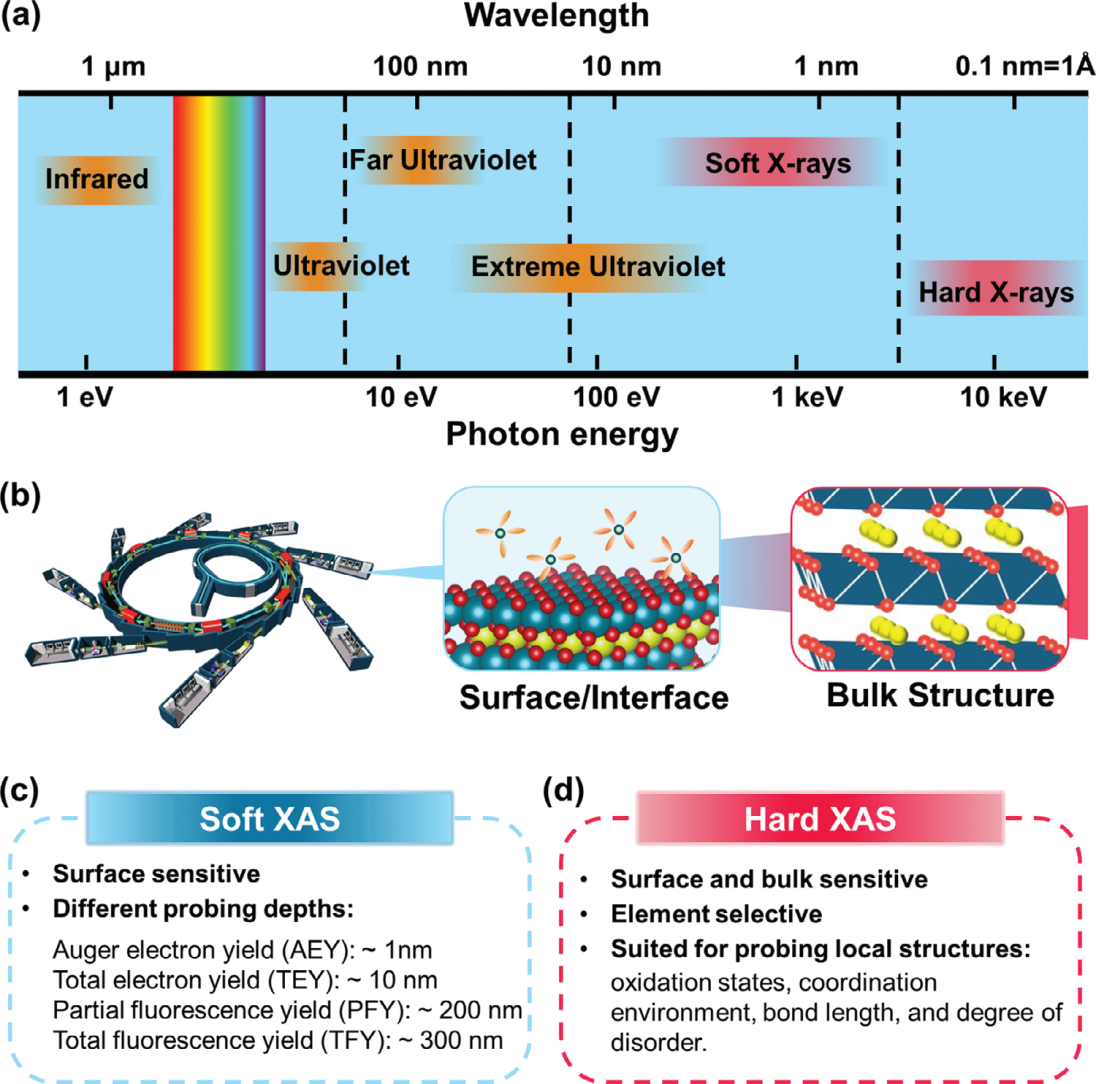
a) Photon energy ranges of synchrotron radiation and the corresponding nominal classification. The gap between “soft” to “hard” X‐ray energies is sometimes defined by the “tender” or “medium” X‐rays. b) Schematic illustration of synchrotron XAS techniques applied on cathode materials study. Summary of the characteristics of soft XAS c) and d) hard XAS techniques.

Synchrotron radiation is generated when electrons traveling at nearly the speed of light, are deflected through strong magnetic fields. As these relativistic, charged particles move through the magnetic field, they lose energy in the form of electromagnetic radiation, including X‐rays.^[^ ^19^ ^]^ Compared to conventional laboratory X‐ray sources, synchrotron X‐rays offer significant advantages, including continuous and tunable photon energy with high brightness and flux across the electromagnetic spectrum, from infrared to X‐rays (**Figure** **1**a). Over the past few decades, synchrotron‐based techniques have been extensively applied to investigate battery materials through ex situ, in situ, and operando experiments. For instance, synchrotron X‐ray diffraction has been widely used to identify the structural properties of crystalline battery materials, particularly cathodes, providing detailed structural information and monitoring dynamic phase transitions during operando studies.^[^ ^20^, ^21^ ^]^ These insights have significantly advanced our fundamental understanding of battery mechanisms. However, the complexity of rechargeable battery systems, which comprise multiple components, such as active materials, conductors, separators, and electrolytes in crystalline, amorphous, and mixed states, requires integrated studies combining scattering, spectroscopy, and imaging techniques to resolve challenges at multiple scales.

Synchrotron XAS is a powerful tool for investigating the electrochemical processes within rechargeable batteries by probing the local electronic structure and coordination environments of specific elements in cathode materials. Unlike other localized techniques, such as transmission electron microscopy (TEM), synchrotron XAS provides statistically reliable information by averaging the local atomic structure over several square millimeters of a sample.

XAS is typically classified into soft and hard XAS based on the photon energy range. Soft XAS generally covers photon energies below 2–3 keV, where measurements are typically conducted under vacuum conditions. In contrast, hard XAS operates at photon energies above 5 keV, allowing measurements to be performed under atmospheric conditions. The exact classification may vary slightly depending on the specific beamline at different synchrotron facilities worldwide.^[^ ^22^, ^23^, ^24^ ^]^ In this review, we define soft XAS as photon energies below 3 keV. The wide photon energy range, from tens of eVs to tens of keV, enables XAS to study cathode materials at various depths, including surface, interface, and bulk (Figure 1b). The advantages of synchrotron XAS in cathode material research can be summarized as follows:

1. Versatile Probing of Local Structures: XAS is effective for studying local structures in materials, regardless of long‐range ordering. Unlike X‐ray diffraction, which is limited to crystalline materials, XAS can probe matter in various states, including crystalline, amorphous, and disordered phases in both solid and liquid forms. This versatility makes XAS an essential complement to X‐ray diffraction and indispensable for monitoring local structural changes in battery materials.
2. Elemental Selectivity: XAS is an element‐selective technique, particularly useful for studying specific elements within compounds containing multiple elements. This selectivity is crucial for identifying redox centers and understanding the redox processes in complex cathode materials, such as high‐entropy sodium‐ion battery cathodes (e.g., O3‐type NaNi_0.12_Cu_0.12_Mg_0.12_Fe_0.15_Co_0.15_Mn_0.1_Ti_0.1_Sn_0.1_Sb_0.04_O_2_^[^ ^25^ ^]^). This capability has made XAS an important tool for studying charge compensation mechanisms in various battery systems, including Li‐ion batteries, Li‐ion batteries, and Li–/Na–S batteries.
3. Multidepth Structural Information: XAS can provide structural information at multiple depths. Hard XAS, with its high penetration capability, offers bulk structure information, while soft XAS, with lower photon energy, is surface‐sensitive. The probing depth varies significantly with different collection modes of soft XAS, such as electron yield (probing a few nanometers) and fluorescence photon yield (probing hundreds of nanometers) (Figure 1c,d). By comparing information collected from hard and soft XAS in different modes, a comprehensive understanding of the cathode material and depth profile can be achieved, such as the cathode electrolyte interphase (CEI) after cycling.^[^ ^26^, ^27^ ^]^
4. Operando Capabilities: The ability to conduct operando experiments is another significant advantage of XAS. Time‐resolved XAS can probe dynamic processes, such as phase transitions during battery charging and discharging, making it particularly useful for studying rate‐dependent structural changes in cathode materials with multiple redox centers.

### 2.2 Fundamentals, Methodologies, and Data Analysis of XAS

Synchrotron XAS is the measurement of the X‐ray absorption coefficient as a function of photon energy for a specific element.^[^ ^19^ ^]^ When the incident X‐ray photon energy exceeds the threshold needed to excite core electrons of the target element, a significant increase in absorption occurs, known as the absorption edge. An XAS energy spectrum is obtained by tuning the photon energy using a crystal or plane grating monochromator.

Hard XAS, which operates at high photon energy ranges, can penetrate deeper into samples—ranging from micrometers to millimeters—thereby providing bulk information. This makes hard XAS particularly useful for investigating reactions in battery materials under various conditions. Operando hard XAS experiments have been successfully applied to studying electrode materials during charge/discharge cycling and under variable temperatures.^[^ ^16^, ^28^, ^29^, ^30^ ^]^

Hard XAS data are typically collected using either transmission or fluorescence detection modes.^[^ ^31^ ^]^ In transmission mode, the incident X‐ray beam passes through the sample, and the intensities of the incoming and outgoing X‐ray beams are recorded as *I*_0_ and *I*_t_, respectively, commonly using ionization chambers.^[^ ^19^, ^32^ ^]^ The absorption coefficient *μ* of the sample can be calculated using the Bouguer–Lambert–Beer law^[^ ^33^ ^]^ (Equation (1))

(1) $$\mu d=\ln\frac{I_{o}}{I_{t}}$$

where *µ* is the X‐ray absorption coefficient and *d* represents the sample thickness.

In a fluorescence mode, the sample is positioned at ≈45° relative to the incoming X‐ray beam, with the fluorescence detector placed at a right angle to the incident X‐rays (45° relative to the sample). The absorption coefficient *μ* in fluorescence mode can be determined by Equation (2)

(2) $$\mu d\propto \;\frac{I_{f}}{I_{t}}$$

where *I*_f_ is the fluorescence intensity. The choice of detection mode depends on the thickness of the sample and the concentration of the target element. Generally, transmission mode is preferred when the element of interest is sufficiently concentrated, and the sample thickness is adjustable, as it offers a higher signal‐to‐noise ratio and reliable bulk information. Fluorescence mode is better suited for cases where the target element is present in low concentrations (typically below 0.5%).

**Figure 2:**
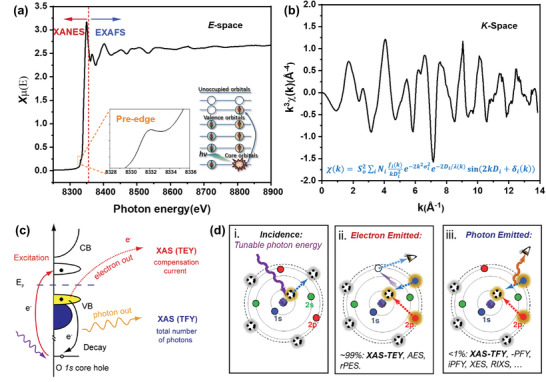
a) Typical XAS spectrum of Ni *K*‐edge in LiNiO_2_ powder. b) *k* ^3^‐weighted Ni *K*‐edge EXAFS oscillations. c) Schematic illustration of the fundamental process and data collection modes of soft XAS (TEY and TFY). Reproduced with permission.^[^ ^27^ ^]^ Copyright 2020, AIP Publishing Portfolio. d) Atomic model of core‐level soft X‐ray absorption and emission. Adapted with permission.^[^ ^34^ ^]^ Copyright 2013, Elsevier. Adapted with permission.^[^ ^35^ ^]^ Copyright 2018, Elsevier.

A full XAS spectrum is generally divided into two regions: X‐ray absorption near‐edge structure (XANES) and extended X‐ray absorption fine structure (EXAFS) (**Figure** **2**a). XANES corresponds to the part of the absorption spectrum within 30–50 eV near the absorption edge energy, while EXAFS begins ≈20–30 eV above the edge energy.^[^ ^32^ ^]^ In soft XAS studies, EXAFS is not typically considered often due to the close presence of additional edges (for example *L*‐ or *M*‐edges). XANES provides critical information on the local electronic and geometric structure around the probed atom.^[^ ^34^ ^]^

In XANES, the energy position of the absorption edge can be used to quantitatively determine the oxidation state of the measured element, shifting to higher energy with increasing oxidation state due to the reduced shielding effect.^[^ ^36^ ^]^ The shape and features of the pre‐edge peaks in the XANES spectrum can be used to identify the local geometric structure, as the interaction between the photon absorber and surrounding ligand atoms may produce pre‐edge or mid‐edge shoulder peaks.^[^ ^37^ ^]^ For 3*d* transition metals, the intensity of pre‐edge peaks varies significantly with the coordination environment (tetrahedral > tetragonal‐pyramidal > octahedral).^[^ ^38^ ^]^ Thus, the XANES region provides valuable insights into the local electronic and geometric structure of the probed atom.

EXAFS, on the other hand, is typically used to probe the radial distribution of neighboring atoms, providing detailed information about atoms within 5–6 Å of the absorbing atom.^[^ ^39^ ^]^ This requires measurement of subtle spectral features, often extending 1000 eV or more above the absorption edge. Unlike the XANES region, which is influenced by electronic transitions and multiple scattering processes, EXAFS arises from a single physical mechanism and can be theoretically modeled to compare with experimental data.^[^ ^40^ ^]^

In EXAFS, incident X‐rays at a specific energy are absorbed, exciting core electrons and generating outgoing photoelectron waves (Figure 2b). These waves are partially scattered by surrounding atoms, and the resulting interference pattern (constructive and destructive regions) depends on the path length traveled by the waves. These interactions manifest as oscillations in the absorption coefficient,^[^ ^41^ ^]^ described by Equation (3)

(3) $$\chi \left(k\right)=S_{o}^{2}\;\sum_{i}N_{i}\frac{f_{i}\left(k\right)}{kD_{i}^{2}}e^{-2k^{2}\sigma_{i}^{2}}e^{-2D_{i}/\lambda \left(k\right)}\sin\left(2kD_{i}+\delta_{i}\left(k\right)\right)$$

where $S_{o}^{2}$ is an amplitude reduction factor, which is directly influenced by shake‐up and shake‐off processes occurring at the central (absorbing) atom because these processes can reduce the effective number of photoelectrons contributing to the observed EXAFS oscillations.^[^ ^42^ ^]^ *f*(*k*) and δ(*k*) represent the scattering amplitude and phase shift of the surrounding atoms, respectively. λ(*k*) is the mean free path of the photoelectron.^[^ ^41^ ^]^ Constructive and destructive interaction is ultimately dependent on the atomic structure, including coordination numbers (*N*), bond lengths (*D*), and bond length disorder (Debye–Waller factor;  σ^2^). By modeling and fitting of the EXAFS spectrum, these parameters can be quantified. Therefore, EXAFS enables a detailed and quantitative analysis of the local atomic structure of the probed material. Additionally, the scattering factors in EXAFS analysis depend on the atomic number (Z) of neighboring atom, making XAS an element‐specific and highly sensitive technique for identifying neighboring atomic species.

Unlike the hard XAS, the penetration depth of soft X‐rays is limited due to their relatively low photon energy, meaning that direct measurement of X‐ray attenuation is typically only feasible for ultrathin samples. Soft XAS spectra of battery materials are generally collected by detecting various decay signals of excited core electrons,^[^ ^24^ ^]^ such as total electron yield (TEY) and total fluorescence photon yield (TFY) (Figure 2c).^[^ ^27^ ^]^ Specifically, a core‐level electron is excited into unoccupied electronic states above the Fermi level by a tunable incident X‐ray (Figure 2d‐i). The excited state then decays, filling the core hole and releasing the absorbed energy by emitting electrons (≈90%, Figure 2d‐ii) or photons (<1%, Figure 2d‐iii).^[^ ^34^, ^35^ ^]^ The TEY mode probes only a few nanometers deep due to the short escape length of electrons,^[^ ^43^ ^]^ while the TFY mode can reach hundreds of nanometers, depending on photon energy.^[^ ^28^, ^44^ ^]^ The simultaneous collection of both TEY and TFY data is possible in most soft XAS systems, allowing for comparison at different depths within the cathode materials.

It is important to note that both TEY and TFY data are derived from secondary X‐ray excitation and relaxation signals. In TEY mode, the sample drain current is typically measured,^[^ ^45^ ^]^ making it suitable only for conductive materials. For TFY mode, a self‐absorption effect should be considered, as this effect results from a sharp change in penetration depth when the incident X‐rays scan through an absorption edge.^[^ ^46^ ^]^ The TMs commonly used in Li‐ion and Na‐ion cathodes, such as Mn, Fe, Cr, are prone to significant spectral line shape distortions at their *L*‐edge spectra in TFY mode due to substantial interference from the O *K*‐edge. This issue contrasts with their respective higher energy *K*‐edges, and with, for instance, the Ni *L*‐edge, which remains largely unattached due to its greater energy separation from the O *K*‐edge. This phenomenon has been observed in various cathode materials, such as LiNi₀.₅Mn₁.₅O₂, LiFePO₄, and Li₀.₃₅Mn₀.₆₅Cr₀.₃₅O₂.^[^ ^47^ ^]^ Therefore, caution is needed when using TFY mode to probe these elements in cathode materials. Partial fluorescence yield (PFY) and inverse partial fluorescence yield (IPFY) modes may help reduce self‐absorption effects and spectral line distortions; however, the underlying mechanisms of PFY and IPFY remain disputed, necessitating further research to clarify their effects.^[^ ^47^, ^48^ ^]^

Beyond TEY and FY modes, resonant inelastic X‐ray scattering (RIXS) has rapidly established itself as a complementary X‐ray technique for studying battery cathodes.^[^ ^49^ ^]^ By recording the energy distribution of emitted photons, RIXS can differentiate between the oxidation states of lattice oxygen in oxide cathodes.^[^ ^50^, ^51^ ^]^ In conventional soft XAS spectra, signals from oxidized oxygen often overlap with those from transition metals due to TM‐O hybridization. In such cases, RIXS serves as an ideal complementary technique for investigating oxygen redox mechanisms, and readers are encouraged to refer to related review articles for more detailed discussions on the fundamentals and applications of RIXS.^[^ ^35^, ^52^ ^]^

The methods and fundamentals of data analysis for ex situ XAS have been comprehensively summarized in our previous review.^[^ ^47^ ^]^ Commonly used software tools, such as Larch, Athena, and Artemis, are widely employed for data processing, analysis, and fitting. However, operando XAS experiments conducted over a single electrochemical cycle can generate tens to hundreds of spectra. A spectrum‐by‐spectrum processing approach for handling such large and complex datasets is not only labor‐intensive but also poses challenges in extracting reliable information. Multivariate Curve Resolution–Alternating Least Squares (MCR–ALS), an alternative and highly effective method, has been successfully applied to analyze extensive XAS datasets.^[^ ^53^ ^]^ MCR is a well‐established chemometric tool, designed to decompose complex mixtures into pure component contributions, yielding matrices that represent physically or chemically meaningful features.^[^ ^54^ ^]^ MCR–ALS, one of the most widely used MCR algorithms, was first proposed by Tauler in 1995^[^ ^55^ ^]^ and has been employed in battery materials research since its first application in 2007.^[^ ^56^ ^]^ This method enables the extraction of unbiased, chemically meaningful components from operando XAS datasets, significantly reducing analysis time and facilitating the identification of intermediate species. Specific applications of MCR–ALS will be discussed in detail in Section 3.1. For further fundamentals of XAS technology, readers may refer to other excellent reviews.^[^ ^7^, ^14^, ^15^ ^]^

### 2.3 Operando XAS Experimental Setup and Cell Design

Several key considerations must be taken into account when performing operando XAS experiments. For hard XAS, operando experiments—whether in transmission or fluorescence mode—require the careful design of apertured cells and the selection of appropriate window materials. In fluorescence mode, the cell is positioned at a 45° angle to the incident beam, necessitating larger apertures to prevent the beam from striking the metallic walls of the aperture.

Additionally, the window materials covering the apertures must provide sufficient X‐ray transmission and possess the mechanical strength necessary to maintain the integrity of the cells during operation. Low‐*Z* window materials, such as polymer films (e.g., Kapton), are preferred due to their low atomic number and minimal X‐ray absorption.^[^ ^57^, ^58^ ^]^ Although beryllium, with its low‐*Z* properties, used to be adopted as X‐ray transparent window materials,^[^ ^59^ ^]^ it is toxic when oxidized. Our group demonstrated that using aluminum or copper tape as window covers is effective for both operando X‐ray diffraction and XAS experiments.^[^ ^60^ ^]^ These covers offer good conductivity and sealing, improving battery performance, especially under high‐voltage conditions. However, copper tape is unsuitable when the absorption energy of the target element is close to that of copper. For soft XAS, only fluorescence mode is feasible for in situ or operando studies when measuring low‐*Z* elements (e.g., O, S, P, Si), as most photons are absorbed by the electrolyte.

While XAS is a crucial technique for studying the local structure of cathode materials, it is not without challenges. First, beam damage is a significant concern, especially during operando experiments where the sample is exposed to high‐flux radiation for extended periods. Prolonged exposure to high radiation doses can lead to the evaporation or decomposition of electrolytes, which in turn impedes electrochemical reactions in batteries—a phenomenon observed across various battery materials during operando synchrotron experiments.^[^ ^61^ ^]^ Factors such as the total radiation dose per electrochemical cycle, electrode mass loadings, and the properties of the electrodes and electrolytes have been shown to play a critical role in the mechanisms underlying beam‐induced damage.^[^ ^62^, ^63^ ^]^ Second, poorly designed in situ cells can lead to unreliable results, particularly under nonequilibrium conditions, such as fast electrochemical cycling. In this manuscript, in situ cells refers to the customized cells designed specifically for conducting operando XAS experiments. Additionally, the longer data acquisition time for XAS measurements, especially for EXAFS, poses challenges in characterizing samples away from equilibrium. Therefore, the development of energy slew scanning techniques that enable rapid collection of full XAS spectra is essential for real‐time monitoring of local structural changes in battery materials under fast charge/discharge rates. Moreover, soft XAS requires ultrahigh vacuum conditions, complicating the design of in situ cells, particularly for systems with liquid electrolytes. Collaboration between battery researchers and beamline scientists is necessary to address these challenges in cell design and operando experiments.

**Figure 3:**
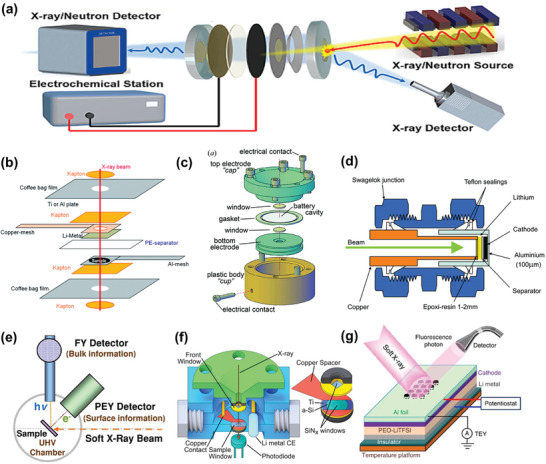
Schematic illustration of electrochemical cells for operando XAS experiments: a–d) In situ hard XAS cells, e) typical soft XAS setup, and f,g) in situ soft XAS cells. a) Coin cell and operando hard XAS experimental setup. Reproduced with permission.^[^ ^29^ ^]^ Copyright 2023, Elsevier. b) “Coffee bag” in situ cell. Reproduced with permission.^[^ ^69^ ^]^ Copyright 2014, Royal Society of Chemistry. c) Argonne's multiple in situ X‐ray (AMPIX) cell. Reproduced with permission.^[^ ^70^ ^]^ Copyright 2021, Wiley‐VCH. d) Swagelok‐type cell. Reproduced with permission.^[^ ^65^ ^]^ Copyright 2005, Wiley‐VCH. e) Typical soft X‐ray experimental setup. Reproduced with permission.^[^ ^28^ ^]^ Copyright 2014, Nature Portfolio. f) Customized cell for operando transmission soft XAS. Reproduced with permission.^[^ ^71^ ^]^ Copyright 2022, Elsevier. g) Customized solid cell with polymer electrolytes for operando soft XAS. Reproduced with permission.^[^ ^72^ ^]^ Copyright 2013, Nature Portfolio.

Additionally, the design of in situ batteries is critical for the successful execution of operando XAS experiments. Although various in situ electrochemical cell designs exist—such as the “Coffee bag” cell, AMPIX cell, and Swagelok cell (**Figure** **3**)—coin cells remain the most widely adopted in XAS experiments due to their ease of assembly and reproducibility. In a typical modified coin cell (Figure 3a),^[^ ^29^ ^]^ holes are punched into the stainless‐steel caps to minimize X‐ray attenuation caused by inactive components, ensuring sufficient X‐rays reach the electrode materials. For hard XAS measurements in transmission mode, two holes on both the positive and negative sides are required, whereas a single hole on the side facing the incident beam may suffice for fluorescence mode (Figure 3a).^[^ ^64^ ^]^ In addition, the stainless‐steel Swagelok‐type cell is widely utilized for operando XAS experiments (Figure 3d).^[^ ^65^, ^66^ ^]^ Its stainless‐steel construction offers excellent chemical resistance and mechanical stability, making it suitable for a wide range of battery materials, electrolytes, and synchrotron experiments.^[^ ^67^, ^68^ ^]^ The cell is also cost‐effective in the long term due to its reusability. However, the high cost of materials and components, coupled with its complex design, can limit accessibility. Incorporating advanced manufacturing techniques, such as 3D printing, in future designs could improve reproducibility and significantly reduce production costs.

These holes are typically sealed with X‐ray transparent materials, such as polyimide (Kapton) film, polyester (Mylar) film, aluminum foil, or beryllium, to prevent electrolyte spills and protect the active materials from air and moisture. However, several key considerations must be made when selecting cover materials. Despite its excellent X‐ray transparency and thermal stability, beryllium should be avoided due to its susceptibility to oxidation during battery charging and the toxicity of both beryllium and its oxides. Kapton film, which is X‐ray transparent and nonhazardous, is a popular choice for hard X‐ray experiments, but it lacks the rigidity needed to ensure tight contact between battery components, potentially leading to delayed reactions, increased impedance, and reduced performance. Additionally, long exposure to high‐energy X‐rays can cause beam damage to Kapton film. Mylar film offers better resistance to chemical attack but has lower X‐ray transparency compared to Kapton.^[^ ^73^ ^]^ Aluminum foil is a lightweight, low‐cost, nonhazardous alternative that is easy to process, making it another widely used window material. When exposed to an X‐ray beam of ≈9 keV, a 10 µm thick aluminum foil achieves over 90% transmission.^[^ ^74^ ^]^ Our previous studies showed that modified coin cells with aluminum foil‐covered holes exhibit better stability than those covered with Kapton film, particularly when charging to high voltages (≈4.9 V).^[^ ^60^ ^]^ It also demonstrates excellent stability in operando XAS experiments for tracking Fe *K* edge evolution (Figure 6a). Thin aluminum foil may serve as a more suitable window material for in situ cells in hard XAS experiments. However, its applicability for studying low‐energy elements, such as V and Ti warrants further investigation.

Soft XAS measurements, due to their relatively low photon energy (typically below 2–3 keV), impose additional requirements on the design of in situ cells, particularly regarding window material selection and thickness. Most window materials suitable for hard XAS, such as thicker Kapton or metallic foils, cause substantial attenuation of the incident X‐rays in the soft X‐ray range. For photon energies below ≈700 eV (e.g., O *K*‐edge or *L*‐edge of transition metals), ultrathin silicon nitride (SiN*_x_*) films (≈50–200 nm) or Kapton films (<5 µm) are preferred due to their high X‐ray transmission and mechanical robustness. At photon energies between ≈1–3 keV (e.g., S *K*‐edge or P *K*‐edge), slightly thicker windows, such as 1–10 µm Kapton films, may be used while maintaining adequate transparency. For soft XAS studies involving vacuum environments or liquid electrolytes, SiN*_x_* films offer superior X‐ray transmission but require careful handling due to their fragility under pressure differentials, whereas Kapton films provide greater mechanical flexibility and impermeability. Optimizing the window material and thickness is critical for minimizing attenuation while ensuring the structural integrity of the in situ cell during operando soft XAS measurements, such as in studies of sulfur cathodes in Na–S batteries.

Soft XAS requires an ultrahigh vacuum (UHV) environment due to its shallow penetration depth (Figure 3e),^[^ ^28^ ^]^ necessitating a different in situ cell design than that used for hard XAS. The limited penetration depth of soft X‐rays generally precludes the use of window materials in in situ cells. In some cases, in situ variable temperature soft XAS has been successfully conducted with an exposed electrode (Figure 3e).^[^ ^28^ ^]^ However, unsealed in situ cells pose potential safety risks, such as electrolyte spills and corrosion, in the UHV environment. A customized liquid in situ cell has been developed for operando transmission soft XAS (Figure 3f), using two 50 nm thick SiN*_x_* membranes as X‐ray transparent covers to prevent electrolyte leakage. Adopting solid‐state electrolytes instead of conventional liquid ones may also offer a feasible solution to these technical challenges. One such in situ cell design for soft XAS incorporates a gel electrolyte, a Li film anode, and the target cathode material on modified aluminum foil with 50‐µm diameter probing holes drilled by laser cutting (Figure 3g).^[^ ^72^ ^]^ These specifically designed holes allow soft X‐rays to reach the cathode materials directly while maintaining normal battery operation. Cell designs may require further customization to accommodate different target elements and battery systems for operando soft XAS experiments. These variations will be discussed in greater detail in Section 3.2.

In summary, when designing in situ cells, minimizing or avoiding interference from inactive components with the incident X‐ray beam is essential. Suitable detection windows and window materials should be selected based on beam size, photon energy, collection modes, and the measurement environment. An ideal window material should offer high X‐ray transparency at the target energy range, chemical and electrochemical stability, impermeability to air and moisture, sufficient pressure resistance, and nonhazardous properties. Low‐Z elements are preferred for other essential battery components, such as current collectors, to reduce X‐ray interaction and ensure a high signal‐to‐noise ratio. For instance, titanium can replace conventional copper foil anode current collectors, which cause significant X‐ray attenuation and degrade XAS data quality. Additionally, continuous exposure to high‐energy X‐rays can lead to severe beam damage, causing abnormal electrochemical reactions or preventing target element reactions altogether.^[^ ^75^ ^]^ High X‐ray doses (typically exceeding 10^1^ MGy) can induce artificial phase transitions in common NMC cathode materials, such as LiNi_0.6_Mn_0.2_Co_0.2_O_2_ and LiNi_0.8_Mn_0.1_Co_0.1_O_2_.^[^ ^63^ ^]^ Controlling the total radiation dose and dose rate has been recognized as crucial for mitigating beam damage.^[58]^ However, feasible dose and dose rate thresholds vary depending on factors such as the electrode material, mass loading, electrode volume, and the properties of other battery components. This variability makes establishing clear dose guidelines challenging. Nevertheless, intermittent data acquisition with low flux density can be an effective strategy to mitigate potential beam damage during operando XAS measurements.

## 3 Applications of Operando XAS for Studying Cathode Materials

Cathode materials are the cornerstone of rechargeable batteries, fundamentally determining the capacity ceiling. These materials are typically classified into two categories based on their reaction mechanisms: intercalation and conversion.^[^ ^76^ ^]^ Intercalation‐type cathode materials contain electrochemically active TM ions as redox centers and alkali ions (e.g., Li and Na) as charge carriers.^[^ ^77^ ^]^ Understanding the valence states and electronic structures of these materials is essential for analyzing redox processes and enhancing battery performance.

In intercalation‐type cathodes, TMs are the primary redox‐active elements responsible for charge compensation. In some cases, additional reversible capacity can be achieved through anionic redox reactions, such as lattice oxygen redox in layered oxide cathodes for Li‐ion batteries or Na‐ion batteries,^[^ ^78^, ^79^ ^]^ and the conversion between sulfur and polysulfides in Li–S or Na–S batteries.^[^ ^80^ ^]^ The redox chemistry of the active elements plays a decisive role in the electrochemical performance of cathode materials. Therefore, element‐selective XAS is indispensable for studying the redox mechanisms and local structure of these materials. This section summarizes the application of operando XAS measurements on different cathode materials and discusses recent advancements in this field.

### 3.1 Operando Hard XAS Studies on Cathode Materials

XAS is suited for probing different elements and edges, depending on the X‐ray energy levels. Hard XAS is commonly employed to measure the *K*‐edge of TMs, such as Mn, Fe, Co, Ni, Cu, and Ru,^[^ ^29^, ^30^, ^81^, ^82^ ^]^ which are the primary redox centers in intercalation‐type cathode materials. This makes hard XAS an ideal technique for investigating redox reaction processes, decoupling concomitant reactions, and probing local structural changes nondestructively.

Among the various intercalation‐type cathodes, oxide cathodes—including layered, spinel, and polyanion oxides—are the predominant classes used in practical Li‐ion batteries.^[^ ^83^ ^]^ For Na‐ion batteries, Prussian blue and its analogues, alongside layered and polyanion oxides, have also garnered significant attention.^[^ ^84^ ^]^ This section reviews operando hard XAS studies categorized by their application to different types of cathode materials.

#### 3.1.1 Layered Oxide Cathodes

**Figure 4:**
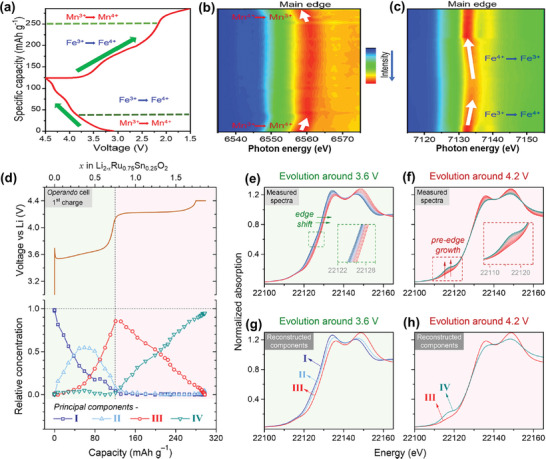
Layered oxide cathodes. a) Corresponding galvanostatic charge/discharge curves of Na||NaFe_0.5_Mn_0.5_O_2_ cell during the operando XAS experiment. 2D contour plots of b) Mn and c) Fe *K*‐edge XANES spectra. Reproduced with permission.^[^ ^29^ ^]^ Copyright 2023, Elsevier. d) Galvanostatic charge voltage profile of Li_2_Ru_0.75_Sn_0.25_O_3_ recorded during operando XAS measurement (the above) and relative concentrations of the four principal components restructured using MCR‐ALS method (the bottom). Operando Ru *K*‐edge XANES spectra during charging at around e) 3.6 V and f) 4.2 V. Reconstructed XANES spectra for g) component I, II, and III, and h) component III and IV. Reproduced with permission.^[^ ^30^ ^]^ Copyright 2017, American Chemical Society.

Layered oxides represent a typical intercalation cathode type, where the structure maintains charge balance through TM redox reactions, despite the repeated insertion/extraction of alkali ions. This process often results in significant structural changes or phase transitions, leading to degradation and reduced cycle life. Common layered oxide cathodes for Li‐ion batteries, such as LiCoO₂, LiNi_1/3_Mn_1/3_Co_1/3_O₂, and LiNi₀.₈Co₀.₁Mn₀.₁O₂, typically undergo a two‐phase reaction involving two hexagonal O3 phases (H1, H2), as demonstrated by operando XRD studies.^[^ ^49^, ^85^ ^]^ Differences in phase transition behaviors have been observed at high charging rates (30 C and 60 C) compared to those at low current densities.^[^ ^86^ ^]^ For instance, quick XAS (Q‐XAS) with a temporal resolution of 2 s was used to investigate the charge compensation mechanism in LiNi_1/3_Mn_1/3_Co_1/3_O₂ at high charging rates. In situ Q‐XAS results showed a continuous shift in the Ni *K*‐edge at a charging rate of 30 C, while the Co *K*‐edge and Mn *K*‐edge spectra remained unchanged, indicating that Ni maintains fast redox kinetics at high rates, whereas Mn and Co redox activities are more rate‐dependent. This suggests that increasing Ni content in layered oxide cathodes could enhance their rate capability. Operando XAS has also proven effective for studying the dynamic charge compensation mechanisms in sodium‐layered oxide cathodes. For example, an operando XAS study on O3‐type NaFe₀.₅Mn₀.₅O₂ demonstrated that Mn^3^⁺/Mn⁴⁺ redox predominantly occurs at low voltages, while Fe^3^⁺/Fe⁴⁺ redox dominates at high voltages (**Figure** **4**a–c).^[^ ^29^ ^]^

Operando XAS is not only a powerful tool for tracking redox reactions of active ions in cathode materials but also provides deep insights by decoupling the contributions of different redox processes and visualizing electrochemical reaction pathways. For example, MCR–ALS analysis of the Ru *K*‐edge spectra in Li₂Ru₀.₇₅Sn₀.₂₅O₃ revealed four orthogonal components (Figure 4d–h).^[^ ^30^ ^]^ The cationic redox (Ru⁴⁺/Ru⁵⁺) at the first voltage plateau (≈3.6 V) involves three components (I, II, and III), while the lattice oxygen redox at ≈4.2 V involves two components (III and IV) (Figure 4d). The Ru *K*‐edge spectra evolve differently during cationic and anionic redox processes (Figure 4e,f). A significant shift toward higher energy occurs at ≈3.6 V, indicating Ru⁴⁺/Ru⁵⁺ cationic redox, while no additional edge shift but a gradual pre‐edge peak growth is observed at ≈4.2 V, demonstrating that Ru⁵⁺ remains unchanged and O^2^⁻/O*ⁿ*⁻ (*n* < 2) redox occurs. The four reconstructed components account for nearly all variations in the experimental XAS data (Figure 4g,h). The evolution of the relative concentrations of these components during delithiation reveals two distinct regimes during charging (Figure 4d), corresponding to cationic and anionic redox, respectively. Furthermore, EXAFS analysis of the reconstructed components tracked local structural variations in Li₂Ru₀.₇₅Sn₀.₂₅O₃. The analysis showed that the structural order characterized by six Ru*─*O bonds, two long Ru*─*M bonds, and one short Ru*─*M bond is preserved during cationic redox, whereas an irreversible distortion of the RuO₆ octahedral coordination is observed during anionic redox. This irreversibility accounts for the distinct first activation process commonly seen in Li‐rich oxide cathodes during the initial cycle compared to subsequent cycles.^[^ ^87^, ^88^ ^]^

Operando EXAFS characterization is also a highly effective method for monitoring dynamic local structure changes in layered oxide cathode materials during charge/discharge cycles, quantitatively estimating lattice distortion, and investigating structural reversibility. Ultrahigh‐Ni layered oxide cathodes (Ni ≥ 0.9) exhibit notably high energy densities, making them among the most promising candidates for next‐generation battery systems.^[^ ^89^ ^]^ However, these cathodes suffer from persistent structural fatigue issues, including layered‐to‐spinel/rock salt phase transformation, TM ion dissolution, and lattice oxygen loss. These issues stem from the high reactivity of Ni at a high state of charge (SoC) > 80% and the Jahn–Teller distortion associated with active Ni^3^⁺. This underscores the importance of understanding local environment variations of Ni in ultrahigh‐Ni cathodes at different SoCs.

**Figure 5:**
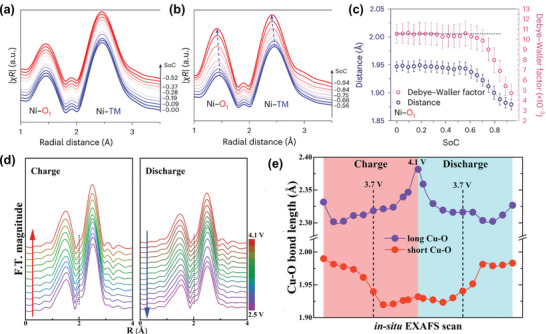
Operando EXAFS study for layered oxide cathodes. Fourier transformed Ni *K*‐edge EXAFS spectra of surface modified LiNi_0.9_Co_0.05_Mn_0.05_O_2_ during the 1st cycle when a) 0 < SoC < 0.52 and b) 0.56 < SoC < 0.94, atomic units and c) the corresponding fitting results of Ni*─*O bond. Reproduced with permission.^[^ ^91^ ^]^ Copyright 2024, Nature Portfolio. d) Fourier transformed Cu *K*‐edge EXAFS spectra of P3‐type Na_2/3_Cu_1/3_Mn_2/3_O_2_ during the charge and discharge process between 2.5 and 4.1 V. e) Comparison of long and short Cu*─*O bond length evolution. Reproduced with permission.^[^ ^81^ ^]^ Copyright 2023, Nature Portfolio.

Fourier transforms based on operando EXAFS data can reveal coordination, bond‐length, and disorder variations through charge cycles.^[^ ^90^ ^]^ A study on epitaxial entropy‐assisted surface‐coated LiNi₀.₉Co₀.₀₅Mn₀.₀₅O₂ (denoted as EEC‐Ni90) demonstrated that Ni*─*O_1_ and Ni*─*TM bond lengths gradually shorten as the SoC decreases below 0.52. Further charging results in a rapid decrease in bond lengths (**Figure** **5**a,b).^[^ ^91^ ^]^ EXAFS fitting can provide quantitative insights into dynamic local structure changes. The Debye–Waller (disorder) factor (σ^2^) of the Ni*─*O bond, obtained from EXAFS fitting, is primarily associated with Jahn–Teller distortions rather than thermal motion or effective nuclear charge alteration via redox processes, making it a useful indicator of Ni^3^⁺ population in the cathode material.^[^ ^92^ ^]^ The change in σ^2^ for the Ni*─*O_1_ bond is consistent with the corresponding bond length change (Figure 5c), indicating no significant accumulation of Jahn–Teller active Ni^3^⁺ at high SoC.^[^ ^91^ ^]^

Liu et al. investigated the dynamic Cu*─*O bond length variations in P3‐type Na_2/3_Cu_1/3_Mn_2/3_O_2_ using operando hard XAS (Figure 5d,e),^[^ ^81^ ^]^ uncovering two distinct charge compensation mechanisms and the origin of stable oxygen redox. Contrary to the conventional expectation of Cu^3^⁺ formation,^[^ ^93^, ^94^ ^]^ their study revealed that the Jahn–Teller distortion of the octahedral CuO₆ unit is preserved throughout electrochemical cycling. Upon charging to high voltage, the significantly elongated Cu*─*O bonds along the *z*‐axis create a local tetrahedral CuO₄‐like environment, akin to a Zhang–Rice‐like singlet state.^[^ ^95^, ^96^ ^]^ This unique spin‐singlet state can stabilize an active oxygen hole, thereby enabling a reversible oxygen redox process.^[^ ^81^ ^]^

In conclusion, operando XAS is valuable for investigating layered oxide cathodes, enabling real‐time analysis of redox mechanisms, phase transitions, and structural changes during battery operation. It can reveal distinct charge compensation behaviors, track dynamic local structure variations, and uncover critical degradation processes like phase transformation and TM ion dissolution, particularly in high‐energy‐density ultrahigh‐Ni cathodes. Additionally, operando XAS has provided key insights into reversible oxygen redox processes, enhancing our understanding of the stability and performance of these materials. This makes operando XAS indispensable for advancing layered oxide cathode design in next‐generation batteries.

#### 3.1.2 Polyanionic Oxide Cathodes

**Figure 6:**
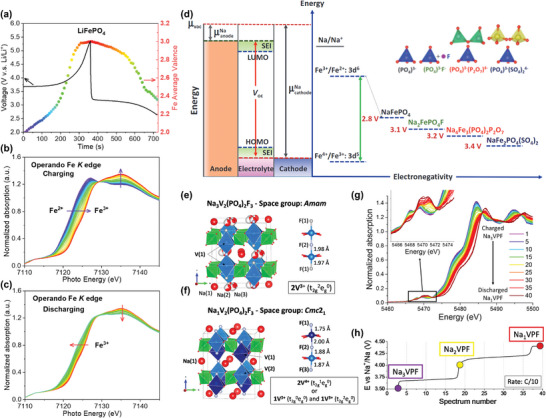
Polyanionic oxide cathodes. a) Galvanostatic charge curve of LiFePO_4_ at 10C and corresponding Fe average valence evolution obtained from operando Fe *K*‐edge XANES spectra during b) charge and c) discharge. d) Schematic illustration of the energy diagram of Fe‐based polyanion cathode materials for Na‐ion batteries. Reproduced with permission.^[^ ^100^ ^]^ Copyright 2019, Wiley‐VCH. Schematic representation of e) Na_3_V_2_(PO_4_)_2_F_3_ and f) Na_1_V_2_(PO_4_)_2_F_3_ with Na atoms in red, VO_4_F_2_ octahedra in blue, and PO_4_ tetrahedra in green. g) Operando V K‐edge XANES spectra collected during the charge, where the inset is the zoom‐in of the pre‐edge region. h) Corresponding galvanostatic charge curve recorded at 0.1C during operando V K‐edge measurements. Reproduced with permission.^[^ ^101^ ^]^ Copyright 2017, American Chemical Society.

In addition to 2D layered TM oxide cathodes, polyanionic oxides with a 3D framework are also prominent insertion‐type cathode materials for rechargeable batteries. Polyanion units, such as (PO_4_)^3−^, (SO_4_)^2−^, (BO_3_)^3−^, (SiO_4_)^4−^, (P_2_O_7_)^4−^, have been widely explored, especially for Li‐ion battery cathodes.^[^ ^97^, ^98^ ^]^ These light and small polyanion units, along with other single anions like F⁻, OH⁻, and N₃⁻, offer significant benefits to batteries by: i) increasing redox potential compared to simple oxides with identical redox elements, and ii) enhancing structural stability and battery safety (**Figure** **6**d).^[^ ^99^ ^]^ The commercial success of olivine LiFePO₄ in the Li‐ion battery market underscores the great potential of polyanion compounds.

XAS characterization has been extensively employed to study the electronic and geometric structure changes in polyanionic oxides.^[^ ^101^, ^102^, ^103^ ^]^ The redox mechanisms of specific redox couples, such asV^3+^/V^4+^,^[^ ^101^ ^]^ V^3.2+^/V^4.2+^,^[^ ^104^ ^]^ and Fe^2+^/Fe^3+^,^[^ ^105^ ^]^ can be directly identified by analyzing the energy position shifts in operando XANES spectra. For example, the evolution of Fe valence in commercial LiFePO_4_ during high‐rate charge/discharge cycles (10 C) has been successfully captured through ultrafast XAS scans (10 s per scan) by our team at the XAS beamline of the Australian Synchrotron (Figure 6a–c).

Furthermore, variations in the pre‐edge features of XANES spectra, related to forbidden 1s to 3*d* quadrupolar transitions facilitated by 3*d*‐*p* orbital hybridization, provide critical insights into the electronic structure and local environment of the probed element.^[^ ^37^ ^]^ For instance, an operando vanadium (V) *K*‐edge XANES study on Na₃V₂(PO₄)₂F₃—a highly promising polyanionic‐based electrode material due to its two high redox voltage plateaus (3.7 and 4.2 V vs Na⁺/Na)—revealed charge disproportionation in the V state (Figure 6e–f).^[^ ^101^ ^]^ The increased intensity of the pre‐edge peak, along with a shift in the main absorption edge around 5487 eV toward higher energy during charging (Figure 6g), indicates V oxidation. To further analyze the data, principal component analysis (PCA)^[^ ^106^ ^]^ was employed to determine the number of principal components contributing to the operando XAS data, followed by MCR‐ALS analysis for stepwise spectral reconstruction. The results showed that two V ions in a bioctahedral unit evolve from V^3^⁺‐V^3^⁺ to V^3^⁺‐V⁴⁺ and finally to V⁴⁺‐V⁵⁺ during the electrochemical extraction of Na⁺ (Figure 6h).

#### 3.1.3 Prussian Blue Analogue Cathodes

**Figure 7:**
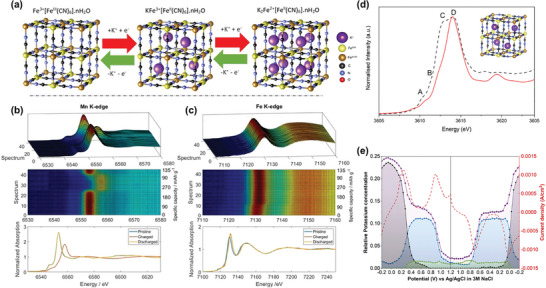
Prussian blue analogue cathodes. a) Schematic illustration of the structural evolution of ideal Prussian blue cathodes during charge carriers (e.g., K^+^) insertion/extraction in an electrochemical cycle. Reproduced with permission.^[^ ^111^ ^]^ Copyright 2023, Royal Society of Chemistry. b) Operando XANES spectra at b) Mn and c) Fe K edge in Na_1.9_Mn_1.1_[Fe(CN)_6_]·2.1H_2_O. Reproduced with permission.^[^ ^109^ ^]^ Copyright 2020, Wiley‐VCH. d) Comparison of experimental potassium *K*‐edge XANES (black dash) and simulated *K*‐edge XANES (red line). e) Pseudoconcentration of potassium groups with respect to applied potential and current density (red dash). Total edge step value of potassium XANES (purple); potassium associated with Prussian white structure (black), Prussian blue structure (blue), and aqueous KNO_3_ (green), respectively. Reproduced with permission.^[^ ^111^ ^]^  Copyright 2023, Royal Society of Chemistry.

Prussian blue analogues (PBAs) are a significant class of insertion‐type cathode materials for secondary batteries,^[^ ^107^ ^]^ particularly Na‐ion batteries and K‐ion batteries. These materials are characterized by a porous bimetal‐cyanide framework, ‐Fe‐CN‐M‐NC‐, where M can be Fe, Mn, Co, Ni, Cu, or Zn.^[^ ^108^ ^]^ The general formula is A*_x_* Fe[M(CN)_6_]*_y_* ·*n*H2O, where A denotes Na⁺ or K⁺.^[^ ^109^, ^110^ ^]^ The cyanide bridges between electroactive Fe and M ions create an open structure with available insertion sites, vacancies, and channels (**Figure** **7**a), facilitating ion transport. However, several challenges hinder the widespread adoption and optimization of PBAs, including capacity fading, limited cycle life, low electronic conductivity, and environmental and safety concerns.

Structurally, defects, vacancies, water content, and crystallinity are critical factors that significantly impact the electrochemical properties of PBAs. For example, studies on manganese hexacyanoferrate Na_1.9_Mn_1.1_[Fe(CN)_6_]·2.1H_2_O have shown that adsorbed water can introduce additional, but irreversible, capacity during charging.^[^ ^109^ ^]^ Processing and storing these materials under dry conditions may help avoid excess adsorbed water, but this approach could substantially increase production costs. Given that PBAs are generally synthesized through aqueous methods, completely removing water from their structure is challenging. Notably, the presence of water primarily affects the Fe redox behavior. Operando XANES studies indicate that both Fe and Mn ions in Na_1.9_Mn_1.1_[Fe(CN)_6_]·2.1H_2_O exhibit electroactivity, with clear edge shifts toward higher energy upon charging (Figure 6b,c).^[^ ^109^ ^]^ However, the Fe *K*‐edge shows an irreversible shift in the first 10 spectra, attributed to adsorbed water. The reversible shift of the Mn *K*‐edge highlights the good reversibility of the Mn^2^⁺/Mn^3^⁺ redox reactions, despite the formation of Jahn–Teller‐active Mn^3^⁺ species at the end of charge. This finding warrants further investigation into Mn‐containing PBAs. Additionally, the impact of interstitial/structural water and hydration degree on electrochemical performance also requires further study.

Understanding the intercalation processes of charge carriers in PBAs is challenging due to their structural complexities. XANES theoretical simulations combined with operando experimental XAS measurements have been employed to monitor the dynamic evolution of charge carriers (*K*⁺) within the structure during electrochemical processes.^[^ ^111^ ^]^ The pre‐edge (feature A), white line shoulder (features B and C), and white line (feature D) of the XANES spectra were successfully simulated using a plane‐wave pseudopotential density functional theory (DFT) method (Figure 7d). This approach allows for identifying the location of K⁺ within the structure, considering the presence of water molecules and defects. Furthermore, the potassium *K*‐edge step values can indicate the relative concentration of *K*⁺ within the PBA cathode. Using linear combination fitting (LCF) of the operando experimental XAS data, the fractions of *K*⁺ species—fully intercalated within the Prussian white structure, partially intercalated within the Prussian blue structure, and associated with aqueous KNO₃ electrolytes—can be determined (Figure 7e).

Overall, hard XAS characterization, with its photon energy range suited for probing electroactive TM ions at the higher energy *K*‐edges, has been widely applied to study major intercalation‐type cathodes. Operando XAS, enhanced by advanced analysis and simulation methods, can reveal reaction intermediates, decouple overlapping reactions, determine the relative concentrations of different species, and accurately track local structural evolutions in a noninvasive manner. This makes it an indispensable technique for studying battery mechanisms. While TM redox is crucial for charge compensation, anionic redox reactions are also significant for achieving high‐performance batteries. Lattice oxygen redox is common in oxide cathodes, with irreversible oxygen redox potentially leading to poor battery performance and safety issues, while reversible oxygen redox can provide additional capacity and contribute to high energy density. Additionally, sulfur‐based batteries have attracted significant attention in recent years. Characterizing these light elements using XAS requires relatively low photon energy, typically soft XAS, which will be discussed in the following section.

### 3.2 Operando Soft XAS Studies on Cathode Materials

Compared to hard XAS, soft XAS is a more direct and sensitive technique for probing the electronic states near the Fermi level, providing critical insights into the physical and chemical characteristics of battery materials. These electronic states are crucial for battery performance, as they regulate working potential, electronic/ionic conductivity, reaction mechanisms, and structural transformations.^[^ ^72^ ^]^ Specifically, in most TM oxide cathodes, the TM 3*d* orbitals are primarily responsible for charge compensation, while *p* orbitals are involved in anionic redox reactions, such as sulfur‐polysulfide and O^2−^–O*^n^* ^−^ (*n* < 2) transitions, in Li‐ion and Na‐ion batteries.^[^ ^12^ ^]^

Soft XAS can directly detect the L‐edge (2*p* → 3*d* transition) of 3*d* TMs and the K‐edge (1*s* → *p* transition) of electrochemically active low‐Z elements like oxygen and sulfur,^[^ ^112^, ^113^ ^]^ providing detailed and quantitative information about the electronic structure and reaction mechanisms. Additionally, the energy range of soft XAS covers the *K*‐edge of elements, such as Li, C, F, Na, P, and Si, which are commonly found in electrolytes, conductive additives, and the solid‐electrolyte interphase (SEI).^[^ ^114^, ^115^ ^]^ This capability enables soft XAS to effectively probe the interactions between electrodes and electrolytes at the interface.

#### 3.2.1 Oxide Cathodes

Harnessing lattice oxygen as an additional redox center is a promising approach to overcoming the capacity limitations of conventional layered oxides that rely solely on TM redox contributions. The use of oxygen redox has been actively studied since the early 2010s in Li‐rich layered cathodes for Li‐ion batteries.^[^ ^88^ ^]^ This phenomenon has also been observed in sodium layered cathodes, including both Na‐rich and Na‐deficient structures, such as P2‐type oxides.^[^ ^78^, ^116^ ^]^ The oxygen redox process allows for additional capacity in these oxide cathode materials, providing a new avenue for the development of high‐energy‐density batteries. However, oxygen redox often results in lattice oxygen loss and structural deterioration, raising concerns about the reversibility of this process.^[^ ^20^, ^117^ ^]^ Achieving reversible oxygen redox remains controversial, primarily due to the lack of effective probing methods.

**Figure 8:**
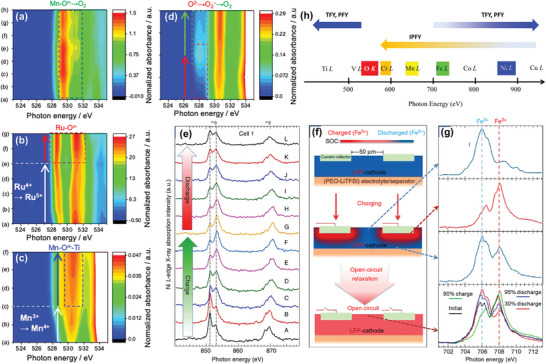
Probing redox mechanisms in oxide cathodes. Operando O *K*‐edge soft XAS spectra of a) Li_1.33_Mn_0.67_O_2_, b) Li_1.33_Ru_0.67_O_2_, c) Li_1.2_Ti_0.4_Mn_0.4_O_2_, and d) Li_1.2_Ti_0.4_Fe_0.4_O_2_ cathodes obtained at different SoC in FY mode. Reproduced with permission.^[^ ^118^ ^]^ Copyright 2019, American Chemical Society. e) Operando Ni *L*‐edge soft XAS spectra of LiNi_1/3_Mn_1/3_Co_1/3_O_2_ cathode collected in TFY mode. f) Schematic of the charging process of LiFePO_4_ cathode. g) Fe *L*‐edge TFY spectra of LiFePO_4_ recorded at different position in the in situ cell and stacked Fe‐*L*_3_ spectra (bottom) after open‐circuit relaxation. Reproduced with permission.^[^ ^72^ ^]^ Copyright 2013, Nature Portfolio. h) Schematic illustration of the element‐dependent FY modes of soft XAS. Reproduced with permission.^[^ ^47^ ^]^ Copyright 2016, AIP Publishing Portfolio.

Operando soft XAS, particularly at the O *K*‐edge, is essential for directly observing electronic structure variations and clarifying redox mechanisms in layered oxide cathodes during electrochemical reactions. Studies on the O *K*‐edge of four Li‐rich cathodes, Li_1.33_Me_0.67_O_2_ (Me = Mn, Ru) and Li_1.2_Ti_0.4_Me_0.4_O_2_ (Me = Mn, Fe), have revealed that the nature of the metal–oxygen chemical bond plays a critical role in achieving reversible oxygen redox (**Figure** **8**a–d).^[^ ^118^ ^]^ The covalent bond of Ru*─*O stabilizes the oxidation of lattice oxygen through the formation of a ligand hole in the Ru 4*d*–O2*p* hybridized orbital, while the strong ionic bond of Ti*─*O stabilizes the oxygen redox by lowering the energy of electrons in the O 2*p* orbitals. This stabilization is identified by the increased intensity of the characteristic peak in the pre‐edge spectra (<533 eV) of the O *K*‐edge (Figure 8b,c), with peaks at 529.2 and 530.7 eV for Li_1.33_Ru_0.67_O_2_ and Li_1.2_Ti_0.4_Mn_0.4_O_2_, respectively. Conversely, a gradually decreased intensity indicates oxygen loss in Li_1.33_Mn_0.67_O_2_ (Figure 8a), while the appearance and subsequent disappearance of a new peak in the O *K*‐edge spectra of Li_1.2_Ti_0.4_Fe_0.4_O_2_ (Figure 8d) suggests the formation of superoxide species followed by oxygen loss.^[^ ^119^ ^]^

The integrated intensity of the O *K*‐edge pre‐edge region has been used to estimate the density of hole states (DOHS) just above the Fermi level in the O 2*p* and TM 3*d* orbitals, serving as an indicator of oxygen redox activity.^[^ ^21^, ^120^, ^121^ ^]^ However, the pre‐edge intensity variation is significantly influenced by the valence state of 3*d* TMs through TM‐O hybridization, making it difficult to directly identify oxygen redox states.^[^ ^78^, ^122^ ^]^ This challenge is addressed by employing mRIXS, which can distinguish oxygen redox features from the dominant TM‐O signals. Fingerprint features from mRIXS spectra have been widely used to confirm the existence of oxygen redox in layered oxide cathodes.^[^ ^123^, ^124^ ^]^ Recent studies have shown that peroxides and O₂ exhibit distinct patterns in high‐resolution RIXS spectra,^[^ ^125^, ^126^ ^]^ highlighting the essential role of RIXS in probing the different products of oxygen redox. However, there are concerns about irradiation damage during RIXS experiments, as heavy X‐ray radiation can eliminate oxidized oxygen features. Thus, developing high‐efficiency RIXS techniques is crucial to reducing exposure time and enabling operando experiments.

Designing in situ cells for operando soft XAS measurements remains challenging due to the short penetration depth of the soft X‐ray beam and the UHV environment required for these experiments. Using a solid‐state electrolyte (SSE) instead of a conventional liquid electrolyte could be an efficient solution to avoid window covers and prevent electrolyte leakage. Operando soft XAS experiments with organic SSEs, such as poly(ethylene oxide) (PEO), have been successfully conducted. For example, Ni *L*‐edge TFY signals from LiNi_1/3_Mn_1/3_Co_1/3_O_2_ were collected in real‐time during electrochemical cycling with PEO and lithium bis(trifluoromethanesulfonyl)imide (LiTFSI) as the SSE.^[^ ^72^ ^]^ The Ni *L*‐edge spectra, arising from the excitation of Ni 2*p* core electrons to the unoccupied 3d orbitals, indicated the evolution of Ni valence (Ni^2^⁺ → Ni⁴⁺) through changes in the intensity ratio of double‐peak features in the Ni‐*L*₃ region (Figure 8e). Conversely, LiFePO_4_ electrodes assembled similarly responded very slowly to the electrochemical charge/discharge process (Figure 8f,g), requiring long relaxation times (26–40 h) to achieve a uniform state of charge (SoC) distribution. Increasing the electrolyte/binder concentration in the LiFePO_4_ cathode may mitigate this relaxation behavior, although it likely depends on intrinsic ion conductivity, necessitating further experimental verification and theoretical simulation.

Inorganic SSEs, such as Ce_0.9_Gd_0.1_O_1.95_,^[^ ^127^ ^]^ and NASICON‐type Li^+^ conductive glass ceramic,^[^ ^128^ ^]^ have also been explored as electrolytes in operando soft XAS experiments for TM oxide cathodes. However, the relatively low ion conductivity of inorganic SSEs requires extremely thin cathode films (tens to hundreds of nanometers) or high‐temperature environments for real‐time soft XAS data collection, presenting challenges in revealing the true structural properties of cathodes under practical conditions. In this context, quasi‐SSEs, which consist of a liquid electrolyte for ion conduction and a mechanically robust solid matrix, may be ideal for in situ cells in operando soft XAS experiments, though further studies are needed.

Finally, the selection of total fluorescence yield (TFY) mode in operando soft XAS experiments must be carefully considered for different target elements. While TFY mode is the most conventional detection method due to its deeper probing depth (>100 nm) compared to TEY mode (<10 nm),^[^ ^28^, ^43^, ^44^ ^]^ its validity strongly depends on the element type and material composition. Significant distortions have been observed in Cr, Mn, and Fe *L*‐edge XAS spectra in TFY mode, primarily due to self‐absorption and saturation effects.^[^ ^47^ ^]^ Li‐ion battery and Na‐ion battery oxide cathodes are frequently affected by these issues due to O *K* fluorescence emission. In contrast, the Ni *L*‐edge, far from the O *K*‐edge, is less affected. Fortunately, IPFY mode has been shown to effectively reduce spectral line shape distortions.^[^ ^129^, ^130^ ^]^ Thus, detection mode selection in soft XAS experiments should be element‐dependent, with IPFY likely more suitable for elements near the O *K*‐edge than conventional TFY or PFY (Figure 8h).^[^ ^47^ ^]^

#### 3.2.2 Sulfur‐Based Cathodes

**Figure 9:**
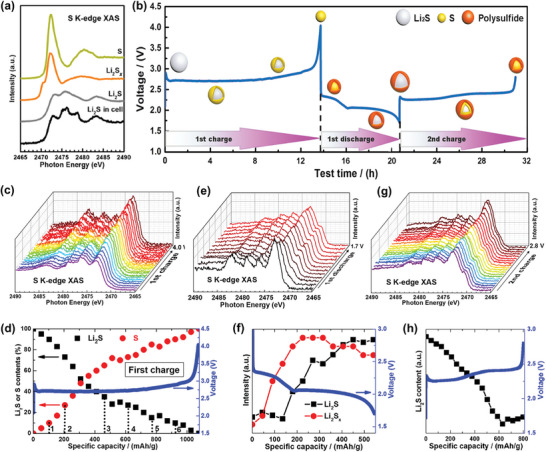
Probing redox mechanisms in S‐based cathodes. a) S *K*‐edge spectra of references (S, Li_2_S_2_, and Li_2_S) and Li_2_S cathode in an in situ cell. b) Reaction mechanism of Li_2_S cathode during the first two charge processes in Li–S batteries. c) Operando S *K*‐edge spectra during initial charge and d) corresponding contents of S and Li_2_S that calculated from two‐phase fitting as a function of specific capacity. d) Operando S *K*‐edge spectra during initial discharge and f) corresponding contents of S and Li_2_S that calculated from two‐phase fitting as a function of specific capacity. g) Operando S *K*‐edge spectra during second charge and h) corresponding contents of Li_2_S as a function of specific capacity. Reproduced with permission.^[^ ^131^ ^]^ Copyright 2017, American Chemical Society.

Similarly, soft XAS can efficiently differentiate various sulfur species (**Figure** **9**a), and has been employed to enhance the understanding of reaction mechanisms in S‐based cathodes for Na–S and Li–S batteries. Li–S and Na–S batteries hold significant potential for large‐scale grid applications and electric vehicles due to their low cost and high theoretical energy densities (2600 Wh kg⁻¹ for Li–S and 1274 Wh kg⁻¹ for Na–S systems).^[^ ^131^, ^132^ ^]^ The performance of these batteries hinges on the sulfur redox reactions in the cathodes, which involve complex conversions between sulfur, Li₂S or Na₂S, and different polysulfides (Figure 9b).^[^ ^131^ ^]^ However, several key challenges—such as the poor conductivity of solid sulfur, high solubility of polysulfides, and sluggish redox kinetics—must be addressed before these batteries can achieve widespread commercialization.

Operando XAS experiments have shown that Li₂S, used as an initial S‐based cathode, directly converts to elemental sulfur via a two‐phase solid–solid reaction during the first charge process (Figure 9c). This is followed by reduction into polysulfides and partial further reduction into Li₂S (Figure 9e). The unreacted polysulfides formed during the initial discharge modify the reaction mechanism in the subsequent charge process: Li₂S is first oxidized to polysulfides and then to elemental sulfur (Figure 9g), indicating a solid–liquid–solid reaction. The evolution of Li₂S, sulfur, and Li_2_S*x* was quantitatively identified by performing two‐phase fitting for the intermediate states using linear combinations of the spectra from the initial and final states (Figure 9d,f,h). Notably, the residual polysulfides play a dual role in electrochemical performance. On one hand, their gradual accumulation results in lower sulfur utilization and a severe “shuttle effect” due to their substantial dissolution into the electrolyte. On the other hand, these polysulfides facilitate the electrochemical transition from Li₂S to polysulfides and enhance charge transfer kinetics at the Li₂S/electrolyte interface. Similar effects have been observed for residual Na₂S in Na–S batteries, where Na₂S, the discharge product, has been confirmed to be partially reversible during electrochemical cycling. Additionally, Na₂S residing in continuous carbon pores serves as a highly Na⁺‐ionic‐conductive interface, significantly reducing Na⁺ migration barriers.^[^ ^132^ ^]^ Thus, tracking the formation and evolution of different sulfur redox intermediate species via operando XAS is essential for deepening our understanding of reaction mechanisms and addressing the existing challenges in S‐based cathodes.

However, it has been noted that sulfur cathodes can suffer severe beam damage from synchrotron X‐ray irradiation. For instance, an X‐ray beam at 7.7 keV can alter the morphology of sulfur, causing it to migrate out of the XAS sampling area, leading to reduced sulfur signal and polysulfide mass transfer artifacts.^[^ ^133^ ^]^ This issue does not cause any potential change under open‐circuit conditions, making it easily overlooked without sampling‐time optimization. Although the incident energy of S *K*‐edge measurement is much lower than 7.7 keV, prolonged X‐ray irradiation and high flux can still cause beam damage to sulfur samples during operando XAS experiments. Future studies should prioritize baseline measurements under open‐circuit conditions to optimize sampling parameters such as beam energy, photon flux, and collection time, ensuring they accurately reflect the true state of S‐based samples.

#### 3.2.3 Interfacial and Surface Chemistry

In addition to electrode research, soft XAS, with its shallow penetration depth, plays a crucial role in interfacial studies. Unlike anodes, where surface parasitic reactions are common,^[^ ^12^, ^134^ ^]^ cathodes generally operate within the electrochemical stability window of electrolytes, resulting in fewer surface reactions under conventional voltage ranges. Early studies of the solid electrolyte interphase (SEI) primarily focused on anodes, as this passivation layer forms due to electrolyte decomposition. However, the development of high‐voltage batteries has brought attention to the SEI of cathodes, often referred to as the cathode electrolyte interphase (CEI).^[^ ^135^ ^]^ The CEI forms due to electrolyte decomposition at high voltages, leading to significant structural changes and transitions in the valence states of TMs at the particle surface. The behavior and properties of these interfaces critically impact the electrochemical performance of both cathodes and anodes, influencing factors, such as rate capability, internal impedance, and capacity retention.^[^ ^27^ ^]^

Soft XAS has proven to be an effective tool for investigating the interfacial compositions, behaviors, and properties of oxide cathodes.^[^ ^135^ ^]^ A common issue in Mn‐based oxide cathodes is Mn dissolution, historically attributed to the disproportionation reaction of Mn^3^⁺ (2Mn^3^⁺ → Mn⁴⁺ + Mn^2^⁺),^[^ ^136^ ^]^ where Mn^2^⁺ is predominantly generated when Mn is in a low valence state at a discharged state. To mitigate this issue, partial substitution with low‐valence elements has been used in Mn‐based cathodes to maintain Mn in a Mn⁴⁺ state, as seen in Ni^2^⁺‐doped LiMn₂O₄ spinel cathodes.^[^ ^137^ ^]^ The LiNi₀.₅Mn₁.₅O₄ cathode, containing only Mn⁴⁺, has gained attention for its high operating voltage (4.7 V), low cost, and good rate capability.

**Figure 10:**
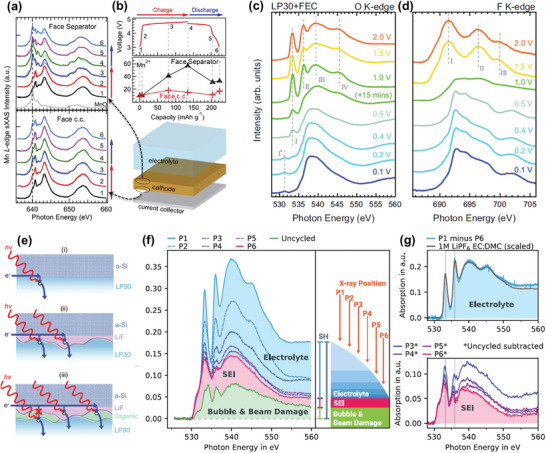
Interfacial and surface research. a) Mn *L*‐edge spectra (TEY) of LiNi_0.5_Mn_0.5_O_4_ cathode surface facing the separator (up) and facing the current collector (down). Reproduced with permission.^[^ ^26^ ^]^ Copyright 2015, Elsevier. Reproduced with permission.^[^ ^27^ ^]^ Copyright 2020, AIP Publishing Portfolio. b) Corresponding voltage profile and with different SoC for soft XAS measurements marked and Mn^2+^ concentration obtained from the simulated results of the Mn spectra in (a). Operando c) O *K*‐edge and d) F *K*‐edge TEY spectra of the SEI formed in the liquid electrolyte, 1 m LiPF_6_ in EC/DMC with FEC additive. e) Schematic representation of the different paths that electrons take to go through the SEI during operando soft XAS experiments in the TEY mode. Reproduced with permission.^[^ ^114^ ^]^ Copyright 2022, Nature Portfolio. f) O *K*‐edge transmission spectra collected at six different sample positions (P1–P6) of an in situ cells with 1 m LiPF_6_ in EC/DMC as electrolytes. The blue, red, and green represents the contributions of the electrolytes, SEI, and bubble and beam damage. g) O *K*‐edge transmission spectra of the electrolytes isolated by subtracting P6 to P1 and O K‐edge spectra of the SEI isolated by subtracting the uncycled spectrum from the cycled spectra (P3–P6). Reproduced with permission.^[^ ^71^ ^]^ Copyright 2022, Royal Society of Chemistry.

However, studies have shown that both LiNi₀.₅Mn₁.₅O₄ and LiMn₂O₄ still suffer from severe Mn dissolution, with the amount of dissolved Mn increasing with the state of charge (SoC). This finding contradicts the traditional understanding based on the disproportionation reaction. Soft XAS characterization of LiNi₀.₅Mn₁.₅O₄ interfaces has clarified the Mn dissolution issue, revealing that Mn^2^⁺ concentrations are significantly higher on the electrode side facing the electrolyte and separator than on the side facing the current collector (**Figure** **10**a,b).^[^ ^26^ ^]^ Moreover, Mn^2^⁺ concentration increases markedly at high charge states, where lattice oxygen loss may occur. Thus, Mn^2^⁺ generation is more closely associated with surface restructuring and electrolyte decomposition side reactions than with disproportionation. While further studies are needed to fully understand the degradation mechanisms of surface structures and electrolytes at high charge states, soft XAS results highlight the importance of improving interfacial stability at high voltages to address Mn loss in Mn‐based oxide cathodes.

Operando soft XAS in TEY mode, with nanoscale interface sensitivity (≈10 nm), can track the chemical evolution of the electrode–electrolyte interphase during electrochemical cycling, providing critical insights into the SEI formation under various operating conditions. Weatherup et al. used operando soft XAS in TEY mode to study the evolution of SEI on O *K*‐edge and F *K*‐edge in a 1 m LiPF₆ in EC/DMC electrolyte with and without FEC additive.^[^ ^114^ ^]^ The distinct peaks in the O *K*‐edge spectra (I‐IV) and F *K*‐edge spectra (I‐III) correspond to the main electrolyte components, such as EC, DMC, and PF₆⁻ ions (Figure 10c,d). The study revealed that LiF formation starts at 1.0 V with the FEC additive, a higher potential than without the additive (< 0.4 V), indicating that defects in the SEI can be passivated by electrolyte decomposition, thereby enhancing interfacial stability. Organic components (*─*(C = O)O*─*) in the SEI form subsequently on top of the inorganic LiF layer (Figure 10e), leading to electronic isolation of the electrode, which renders TEY signals undetectable. In such cases, the more bulk‐sensitive FY detection mode can be used to detect organic SEI components.

When conducting operando XAS experiments, especially for battery systems with liquid electrolytes, the rational design of in situ cells is crucial. In addition to potential leakage issues in vacuum environments, the formation of bubbles in electrolytes under high‐intensity X‐ray irradiation poses a significant concern, potentially introducing artifacts in the collected data. A commonly used solution is the adoption of flow electrolytes to mitigate bubble formation during operando XAS experiments.^[^ ^138^ ^]^ Seidel et al. proposed a novel approach to acquire in situ transmission soft XAS signals of SEI formed under liquid electrolytes.^[^ ^71^ ^]^ By deliberately inducing a gas bubble using high‐intensity X‐rays in a customized in situ cell, excess electrolytes in the beam path are removed, creating an X‐ray transparent electrolyte layer. This approach allows low‐intensity soft X‐rays to pass through the in situ cell (Figure 10f). Consequently, transmission soft XAS spectra of the SEI can be extracted by subtracting the absorption spectra of reference compounds (Figure 10g).

In summary, operando soft XAS can effectively monitor both cationic and anionic redox processes, as well as interfacial properties, by combining TEY and TFY detection modes. This makes soft XAS an indispensable technique for investigating various battery systems. One of the primary challenges is the design of in situ cells suitable for UHV conditions. The rapid development of solid‐state electrolytes (SSEs) and quasi‐SSEs is expected to address the major safety issues associated with in situ cells in vacuum environments, making operando soft XAS more versatile and widely applicable.

## 4 Conclusions and Perspectives

This review underscores the transformative role of operando synchrotron XAS in advancing the understanding of cathode materials in rechargeable batteries. The ability to probe the local electronic structure, coordination environments, and redox states of specific elements with high precision has proven invaluable in elucidating complex electrochemical processes. The application of hard and soft XAS techniques has been critical in studying various cathode materials, including layered oxides, polyanionic compounds, Prussian blue analogues, and sulfur‐based cathodes. These techniques allow for multidepth analysis, ranging from surface interactions to bulk phenomena, providing a comprehensive understanding of charge compensation mechanisms, structural evolution, and the identification of reaction intermediates.

Operando XAS has been particularly effective in monitoring real‐time changes in cathode materials during electrochemical cycling. It has offered unprecedented insights into complex processes, such as oxygen redox, anionic redox mechanisms, and interfacial phenomena. The integration of advanced data analysis techniques, such as MCR‐ALS, has further enhanced the ability to deconvolute overlapping reactions and visualize the evolution of multiple species during battery operation.

Looking ahead, several key areas require focus to fully harness the potential of operando XAS in battery research:

1. Mitigating Beam Damage in Long‐Time Operando Experiments: Prolonged exposure to synchrotron X‐rays can cause beam damage, particularly in delicate materials, such as sulfur‐based cathodes. To prevent sample degradation and ensure accurate real‐time measurements, it is essential to optimize photon flux, beam size, and exposure time. Implementing intermittent data acquisition strategies and developing high‐efficiency detectors will also help mitigate these risks.
2. Theoretical Simulations and Advanced Data Analysis: The integration of theoretical simulations with XAS measurements can significantly enhance the identification of multiple species and reaction intermediates, advancing both qualitative and quantitative analysis. Techniques like MCR‐ALS offer powerful tools to reconstruct real XANES components and their temporal evolution, providing deeper insights into complex electrochemical processes. In addition, modeling spectral features using empirical or ab initio parameters^[^ ^139^ ^]^ offers an alternative approach for understanding intricate spectral data, such as those from multivalent transition metal compounds in batteries.
3. Multidepth Analysis Using Soft X‐ray Techniques: Soft XAS, with varying incident energy levels, holds significant potential for probing surface and subsurface regions of cathode materials. This approach could provide a more nuanced understanding of the interaction between surface chemistry and bulk structure, which is critical for improving cathode stability and performance.
4. Development of Reliable Techniques for Studying Oxygen Redox: While soft XAS is widely used to investigate oxygen redox, challenges, such as beam damage, signal interference, and reproducibility persist. RIXS emerges as a more reliable technique for accurately fingerprinting oxygen redox states. Future research should focus on integrating RIXS with other advanced methodologies to achieve a comprehensive understanding of oxygen behavior across different cathode materials.
5. Enhancing Synchrotron Facilities and In situ Cell Design: Expanding synchrotron facilities capable of performing operando soft XAS experiments is crucial to meet the growing demand for high‐resolution, real‐time studies of battery materials. The design of in situ cells remains a critical challenge, particularly in maintaining ultrahigh vacuum conditions while accommodating liquid electrolytes or solid‐state systems. Collaborative efforts between material scientists, beamline experts, and engineers are essential to develop more effective and versatile in situ cells.
6. Combining with Other Synchrotron‐Based Techniques: Combining XAS with other synchrotron‐based techniques, such as X‐ray diffraction and computed tomography, can achieve multiscale characterization of electrode materials. This integration will provide a comprehensive understanding of both local and long‐range structures. Additionally, incorporating an automated sample changer and faster scanning techniques can facilitate the simultaneous measurement of multiple electrochemical cells. These advancements are crucial for optimizing synchrotron XAS beamtime and achieving high‐throughput measurements.
7. Broadening the Application of Operando XAS: While this review focuses on cathode materials for Li‐ion, Na–ion, Li–S, and Na–S batteries, the methodologies and insights gained from operando XAS can be extended to other emerging battery systems, such as solid‐state, Mg‐ion, and multivalent‐ion batteries. As the energy storage landscape evolves, operando XAS will continue to play a pivotal role in driving innovation and improving the performance of next‐generation battery technologies.

By addressing these challenges and leveraging the strengths of operando XAS, the field of battery research is poised for breakthroughs that will lead to higher energy densities, longer cycle lives, and safer, more reliable energy storage systems.

## Conflict of Interest

The authors declare no conflict of interest.
